# Prevalence thresholds for wasting, overweight and stunting in children under 5 years

**DOI:** 10.1017/S1368980018002434

**Published:** 2018-10-09

**Authors:** Mercedes de Onis, Elaine Borghi, Mary Arimond, Patrick Webb, Trevor Croft, Kuntal Saha, Luz Maria De-Regil, Faith Thuita, Rebecca Heidkamp, Julia Krasevec, Chika Hayashi, Rafael Flores-Ayala

**Affiliations:** 1 Department of Nutrition for Health and Development, World Health Organization, 20 Avenue Appia, CH1211 Geneva 27, Switzerland; 2 Center for Dietary Intake Assessment, Washington, DC, USA; 3 Friedman School of Nutrition Science and Policy, Tufts University, Medford, MA, USA; 4 ICF International, Rockville, MD, USA; 5 Nutrition International, Research and Evaluation, Ottawa, Ontario, Canada; 6 School of Public Health, University of Nairobi, Nairobi, Kenya; 7 Department of International Health, Johns Hopkins Bloomberg School of Public Health, Baltimore, MD, USA; 8 Division of Data, Research and Policy, UNICEF, New York, NY, USA; 9 Division of Nutrition, Physical Activity, and Obesity, Centers for Disease Control and Prevention, Atlanta, GA, USA

**Keywords:** Wasting, Overweight, Stunting, Malnutrition, Children

## Abstract

**Objective:**

Prevalence ranges to classify levels of wasting and stunting have been used since the 1990s for global monitoring of malnutrition. Recent developments prompted a re-examination of existing ranges and development of new ones for childhood overweight. The present paper reports from the WHO–UNICEF Technical Expert Advisory Group on Nutrition Monitoring.

**Design:**

Thresholds were developed in relation to sd of the normative WHO Child Growth Standards. The international definition of ‘normal’ (2 sd below/above the WHO standards median) defines the first threshold, which includes 2·3 % of the area under the normalized distribution. Multipliers of this ‘very low’ level (rounded to 2·5 %) set the basis to establish subsequent thresholds. Country groupings using the thresholds were produced using the most recent set of national surveys.

**Setting:**

One hundred and thirty-four countries.

**Subjects:**

Children under 5 years.

**Results:**

For wasting and overweight, thresholds are: ‘very low’ (<2·5 %), ‘low’ (≈1–2 times 2·5 %), ‘medium’ (≈2–4 times 2·5 %), ‘high’ (≈4–6 times 2·5 %) and ‘very high’ (>≈6 times 2·5 %). For stunting, thresholds are: ‘very low’ (<2·5 %), ‘low’ (≈1–4 times 2·5 %), ‘medium’ (≈4–8 times 2·5 %), ‘high’ (≈8–12 times 2·5 %) and ‘very high’ (>≈12 times 2·5 %).

**Conclusions:**

The proposed thresholds minimize changes and keep coherence across anthropometric indicators. They can be used for descriptive purposes to map countries according to severity levels; by donors and global actors to identify priority countries for action; and by governments to trigger action and target programmes aimed at achieving ‘low’ or ‘very low’ levels. Harmonized terminology will help avoid confusion and promote appropriate interventions.

Prevalence ranges to classify levels of undernutrition have been used since the early 1990s for global monitoring of malnutrition. Recommended ranges to be used for stunting were originally determined based on an analysis of seventy-nine national surveys from low- and middle-income countries by grouping them into four categories corresponding approximately to the observed quartiles: ‘low’ (<20 %); ‘medium’ (20–29 %); ‘high’ (30–39 %); and ‘very high’ (≥40 %)^(^ ^1^ ^)^. For wasting, the prevalence ranges were derived on the basis of the association between the prevalence of low weight-for-height and crude mortality rates (defined as below 80 % of median weight-for-height using the National Center for Health Statistics reference^(^ ^2^ ^)^) among children under 5 years in forty-two refugee camps^(^ ^3^ ^)^: ‘acceptable’ (<5 %); ‘poor’ (5–9 %); ‘serious’ (10–14 %); and ‘critical’ (≥15 %). These prevalence ranges for stunting and wasting were endorsed in 1993 by the WHO Expert Committee on Physical Status: The Use and Interpretation of Anthropometry^(^ ^4^ ^)^ and used widely thereafter. No such prevalence ranges were determined for childhood overweight.

The original terminology used for stunting – ‘classification of prevalence levels’ – sought to describe and categorize the range of prevalence rates across the world. However, over time the use of these levels evolved to carry a meaning of public health significance. For wasting, the original terminology – ‘severity index for malnutrition in emergency situations’ – was related to how the ranges were established based on their association with defined mortality risk.

Recently, several developments motivated a re-examination of the classification of these prevalence levels. First, the ongoing global reduction of stunting is approaching the level currently designated as ‘low’^(^ ^5^ ^)^, with 45 % of countries (sixty out of 134) having stunting rates below 20 %. Second, release of the WHO Child Growth Standards in 2006 changed survey estimates compared with the previously used international growth reference (e.g. increased prevalence of wasting and stunting)^(^ ^6^ ^)^. Third was the need to have a similar classification for overweight, considering its rising prevalence and the inclusion of childhood overweight as one of the Global Nutrition Targets for 2025^(^ ^7^ ^)^. Last, there was a need to reconsider terminology given implications of public health significance that are unjustified because the classification for stunting was not based on any association with functional outcomes. The previous approach simply represented a convenient statistical grouping of survey estimates from different countries available at a given point in time.

WHO and UNICEF jointly established an independent Technical Expert Advisory Group on Nutrition Monitoring (TEAM) to advise on how to improve the quality of nutrition monitoring efforts at all levels (http://www.who.int/nutrition/en/). The TEAM was charged with the task of reconsidering the prevalence levels for stunting and wasting and establishing new ones for overweight. The TEAM was also requested to reconsider current terminology and harmonize the labels employed to refer to the different categories across the three anthropometric indicators.

The TEAM’s view was that any revision should be done cautiously and be well justified to avoid confusion and disruption. Pros and cons should be carefully considered before recommending any change in current practice. The present paper describes the background, technical considerations, methods and results of the TEAM recommendations in implementing its mandate.

## Methods

Members of the TEAM and/or the WHO–UNICEF Secretariat prepared background documentation and ran analyses. These were reviewed at the TEAM’s biannual meetings where decisions were made by consensus. Three approaches were considered: (i) a ‘descriptive approach’ – similar to the method used in the 1990s for stunting – by which prevalence levels are established based on a descriptive analysis grouping the latest nationally representative anthropometric estimates into four categories that correspond to observed quartiles; (ii) a ‘functional approach’ – similar to the method used in the 1990s for wasting – by which prevalence levels are based on association with increased risk of functional outcomes (morbidity/mortality); and (iii) a ‘novel approach’ that would set prevalence levels based on degrees of deviation from normality as defined by the WHO Child Growth Standards (hereafter referred to as the ‘WHO standards’)^(^ ^8^ ^)^.

In deciding which approach to apply, the expert group acknowledged the importance of applying the same methodology for all three indicators. The ‘functional approach’ was unanimously identified as being the conceptually appropriate one. However, the group recognized this approach is currently not feasible for stunting and overweight due to the scarcity of required data sets/studies. Furthermore, different functional outcomes would likely relate differently to different prevalence levels, thereby making linking stunting and overweight with functional outcomes challenging. Additionally, the expert group recognized that establishing a process and developing a research agenda to collect the required evidence would take substantial time, while the revision was considered urgent. Nevertheless, it was made clear that the ‘functional approach’ is the preferred one.

**Fig. 1:**
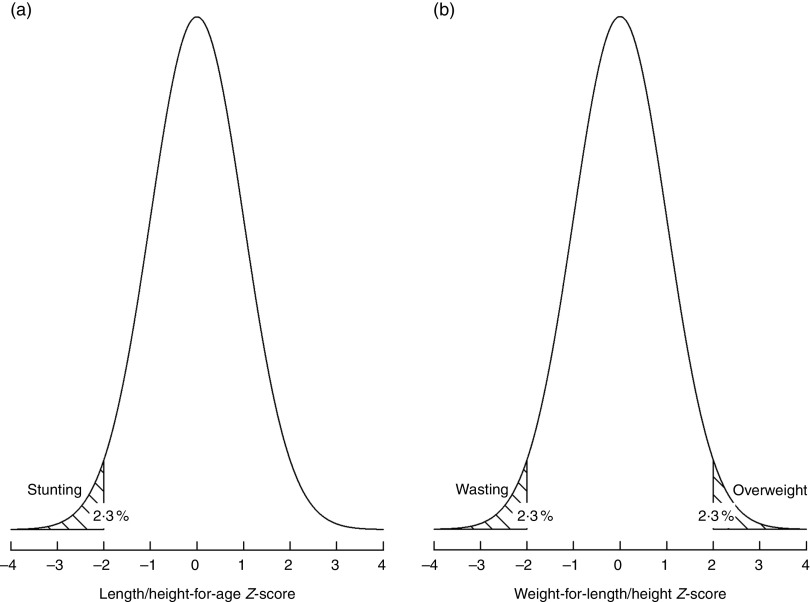
Distribution of the length/height-for-age (a) and weight-for-length/height (b) *Z*-scores in a healthy population: normal distribution with mean=0 and sd=1

When considering the ‘descriptive approach’, the main advantage would be its continuity with the previously used method^(^ ^1^ ^)^; however, using this approach would require redoing the exercise every certain number of years as, for stunting, prevalence levels are trending downwards and will likely continue in that direction^(^ ^5^ ^)^. The TEAM therefore decided to proceed by applying the ‘novel approach’ through which thresholds are defined in relation to sd of the WHO standards distribution. The internationally agreed definition of normality (i.e. 2 sd below or above the WHO standards median)^(^ ^4^ ^)^ defines the first threshold (labelled as ‘very low’ prevalence) which, by definition, includes 2·3 % of the area under the normalized distribution (Fig. 1). Multipliers of this ‘very low’ level (rounded to 2·5 %) set the basis to derive subsequent higher prevalence thresholds as presented below.

To evaluate the implications of the prevalence thresholds established based on the ‘novel approach’, the most recent set of nationally representative surveys from 134 countries including children under 5 years of both sexes^(^ ^9^ ^)^ was used to assess country groupings that would result from applying the new thresholds. Furthermore, for wasting, the plausibility of the new thresholds in relation to the known association of different degrees of severity of wasting with overall childhood mortality was also taken into account.

## Results

Table 1 presents the prevalence thresholds, labels and number of countries in different threshold categories for wasting, overweight and stunting. Labels have been harmonized across indicators as ‘very low’, ‘low’, ‘medium’, ‘high’ and ‘very high’.

**Table 1:** Prevalence thresholds, corresponding labels and number of countries in different prevalence threshold categories for wasting, overweight and stunting in children under 5 years using the ‘novel approach’

Wasting*			Overweight*			Stunting*		
Prevalence thresholds (%)	Labels	No. of countries	Prevalence thresholds (%)	Labels	No. of countries	Prevalence thresholds (%)	Labels	No. of countries
<2·5	Very low	36	<2·5	Very low	18	<2·5	Very low	4
2·5–<5	Low	33	2·5–<5	Low	33	2·5–<10	Low	26
5–<10	Medium	39	5–<10	Medium	50	10–<20	Medium	30
10–<15	High	14	10–<15	High	18	20–<30	High	30
≥15	Very high	10	≥15	Very high	9	≥30 %	Very high	44

* Wasting and overweight available for only 132 and 128 countries, respectively. Stunting available for 134 countries.

For wasting and overweight, the thresholds are the same: ‘very low’ (<2·5 %); ‘low’ (≈1–2 times 2·5 %); ‘medium’ (≈2–4 times 2·5 %); ‘high’ (≈4–6 times 2·5 %); and ‘very high’ (>≈6 times 2·5 %). For stunting, the new thresholds are: ‘very low’ (<2·5 %); ‘low’ (≈1–4 times 2·5 %); ‘medium’ (≈4–8 times 2·5 %); ‘high’ (≈8–12 times 2·5 %) and ‘very high’ (>≈12 times 2·5 %).

## Discussion

The prevalence thresholds presented here for children under 5 years of age were established in relation to sd of the normative WHO standards while keeping in mind the implications for the classification of countries in relation to observed quartiles (e.g. that will not result in an unrealistic distribution of countries such as most or none being classified as ‘high’). The ‘novel approach’ employed is not only conceptually sound but also results in longer-lasting thresholds that will not require updating unless the normative WHO standards are revised.

For wasting (2 sd below the WHO standards weight-for-length/height median)^(^ ^4^ ^)^, the ‘novel approach’ results in similar thresholds to those recommended in the early 1990s^(^ ^4^ ^)^. At that time, the prevalence ranges were derived based on evidence of the association between crude mortality rates and low weight-for-height (<80 % of the National Center for Health Statistics reference median) among children under 5 years in forty-two refugee camps^(^ ^3^ ^)^. The researchers grouped the populations on the basis of their rate of child malnutrition and calculated a weighted average of mortality rates within each group. A progressive increase in mortality rates was found with increasing ranges of child malnutrition rates, indicating, for example, that populations with malnutrition rates in the 5–9·9 % range had mortality rates more than 2·44 times that of populations with malnutrition rates less than 5 %. The original thresholds (i.e. ‘acceptable’ (<5 %); ‘poor’ (5–9 %); ‘serious’ (10–14 %); and ‘critical’ (≥15 %)) have been used for two decades without concerns raised about their applicability in the field. Importantly, they are also in accordance with more recent evidence of the association of wasting with risk of childhood mortality^(^ ^10^ ^)^. When applied to the most recent set of national surveys – where wasting prevalence ranges from 0·1 to 22·4 %^(^ ^9^ ^)^ – the categories provide a distribution of countries (thirty-three, thirty-nine, fourteen and ten for ‘low’, ‘medium’, ‘high’ and ‘very high’, respectively) that is workable for available interventions. Notably, keeping wasting prevalence thresholds untouched preserves programmatic practices from the troubles any change would entail.

Childhood overweight (2 sd above the WHO standards weight-for-length/height median)^(^ ^4^ ^)^ is currently recognized as a global public health problem with important consequences for incidence of acute and chronic diseases, healthy development, and the economic productivity of individuals and societies^(^ ^11^ ^)^. There were no recommendations made in the 1990s for thresholds for overweight prevalence in young children; neither were they made afterwards notwithstanding recognition of this health problem^(^ ^12^ ^,^ ^13^ ^)^. Despite wasting and overweight being nutritional disorders with very distinct causality and preventive/therapeutic interventions^(^ ^14^ ^)^, they nevertheless represent both ends of the same weight-for-length/height distribution and their current prevalence ranges are similar (i.e. overweight prevalence ranges from 0·1 to 26·5 %^(^ ^9^ ^)^). These considerations were the conceptual basis to apply the ‘novel approach’ for overweight using similar multipliers of the WHO standards sd as those used for wasting. The resulting categories provide the following grouping of countries: thirty-three, fifty, eighteen and nine for ‘low’, ‘medium’, ‘high’ and ‘very high’, respectively. Linking programmatic actions^(^ ^7^ ^)^ to the thresholds recommended here will be important to prevent further upward trends and increased risk of overweight and obesity in school-aged children and adolescents^(^ ^15^ ^)^.

Stunting is the most prevalent form of childhood undernutrition, with an estimated 155 million children worldwide falling below 2 sd from the WHO standards length/height-for-age median in 2016^(^ ^5^ ^)^. After years of inattention, stunting is now recognized as a key indicator of overall children’s well-being and a reflection of social inequalities^(^ ^16^ ^,^ ^17^ ^)^. Identified as a major global health priority and the focus of several high-profile initiatives (Scaling Up Nutrition, Zero Hunger Challenge, Nutrition for Growth Summit, African Leaders for Nutrition), stunting is also at the heart of the Global Nutrition Targets for 2025 and the Sustainable Development Goals for 2030. Unfortunately, as with childhood overweight, despite the numerous severe consequences of stunted growth, sufficient data sets are still lacking documenting the association of different degrees of linear growth failure with the pathological disorders for which stunting acts as a biological marker^(^ ^16^ ^)^.

Nevertheless, rigorous evidence demonstrates the association of severe (<−3 sd from the WHO standards median) and moderate (<−3 to <−2 sd from the WHO standards median) stunting with overall child mortality: OR=4·1 (95 % CI 2·6, 6·4) and OR=1·6 (95 % CI 1·3, 2·2), respectively (reference group is more than −1 sd)^(^ ^10^ ^)^. As a comparison, for severe and moderate wasting, OR=9·4 (95 % CI 5·3, 16·8) and OR=3·0 (95 % CI 2·0, 4·5), respectively^(^ ^10^ ^)^. Mindful of the association with mortality, as well as the larger range of stunting prevalence worldwide (1·3 to 55·9 %)^(^ ^9^ ^)^, the ‘novel approach’ was applied doubling the multipliers of normality (i.e. deviation from the WHO standards median) compared with those used for wasting and overweight (Table 1). The resulting classification provides a grouping of countries that is largely in accordance with those that would have been derived if using the ‘descriptive approach’ (i.e. twenty-six, thirty, thirty and forty-four for ‘low’, ‘medium’, ‘high’ and ‘very high’, respectively). Only the ‘very high’ category (≥30 %) includes a larger number of countries (forty-four countries). Of this, twenty-seven have a prevalence between 30 and 39·9 %, fourteen between 40 and 49·9 %, and only three countries have prevalence rates ≥50 % (Timor Leste: 50·2 % in 2013; Eritrea: 50·3 % in 2010; Burundi: 55·9 % in 2016–17)^(^ ^9^ ^)^.

As for wasting and overweight, it will be essential to link each of these prevalence thresholds to programmatic actions/interventions based on evidence currently available to prevent linear growth failure^(^ ^17^ ^,^ ^18^ ^)^ while keeping in mind the context of the double burden of malnutrition that many middle- and low-income countries face^(^ ^19^ ^)^.

Clear and harmonized terminology is important to avoid confusion and promote appropriate actions. On this, the expert group opted for referring to this classification as ‘prevalence thresholds’, a lexis more in line with its intended population-based application; as opposed to ‘cut-offs’, a term mainly used for interpreting measurements of individual children. To harmonize the labels used for the different categories, the TEAM opted to maintain the widely used original labels for stunting as recommended by the WHO Expert Committee on Physical Status: The Use and Interpretation of Anthropometry (‘low’, ‘medium’, ‘high’ and ‘very high’)^(^ ^4^ ^)^ and apply them also to wasting and overweight. Importantly, a category labelled ‘very low’ – that is, of no public health concern – has been added to reflect the expected prevalence of 2·3 % (rounded to 2·5 %) below/above 2 sd from the WHO standards median (Fig. 1).

The revised prevalence thresholds presented here are recommended to replace those in current use – WHO and UNICEF have started using them in their official reporting^(^ ^20^ ^)^ – while minimizing change and keeping coherence across anthropometric indicators. They can be used by the international nutrition community for descriptive purposes in mapping countries according to levels of severity of malnutrition^(^ ^20^ ^)^; by donors and global actors to identify priority countries for action^(^ ^10^ ^,^ ^11^ ^)^; and, most importantly, by governments for monitoring purposes and to trigger informed action and programmes aimed at achieving ‘low’ or ‘very low’ levels. The latter will require recommended actions to be taken at each level for each nutritional disorder. To date only concrete programmatic actions for ‘high’ and ‘very high’ levels of wasting have been recommended in the context of the management of nutrition in major emergencies. Wasting rates of ≥15 % or 10–14 % with aggravating factors require general rations (unless the situation is limited to vulnerable groups), supplementary feeding generalized for all members of vulnerable groups (especially children and pregnant and lactating women) and therapeutic feeding programmes for severely undernourished individuals^(^ ^21^ ^)^. A revision of these actions, based on new programmatic evidence^(^ ^22^ ^)^, and recommendations of others for ‘low’, ‘medium’, ‘high’ and ‘very high’ wasting, overweight and stunting, is a future need.

## References

[1] de Onis M, Monteiro C, Akre J et al. (1993) The worldwide magnitude of protein–energy malnutrition: an overview from the WHO Global Database on Child Growth. Bull World Health Organ 71, 703–712. 8313488 PMC2393544

[2] Dibley MJ, Goldsby JB, Staehling NW et al. (1987) Development of normalized curves for the international growth reference: historical and technical considerations. Am J Clin Nutr 46, 736–748. 3314468 10.1093/ajcn/46.5.736

[3] Nieburg P, Person-Karell B & Toole MJ (1992) Malnutrition–mortality relationships among refugees. J Refug Stud 5, 247–256.

[4] World Health Organization (1995) Physical Status: The Use and Interpretation of Anthropometry. Report of a WHO Expert Committee . WHO Technical Report Series no. 854. Geneva: WHO. 8594834

[5] UNICEF, World Health Organization & World Bank (2017) UNICEF–WHO–World Bank Joint Child Malnutrition Estimates. Key findings of the 2017 edition. http://www.who.int/nutgrowthdb/jme_brochoure2017.pdf?ua=1 (accessed December 2017)

[6] de Onis M, Onyango AW, Borghi E et al. (2006) Comparison of the WHO Child Growth Standards and the NCHS/WHO international growth reference: implications for child health programmes. Public Health Nutr 9, 942–947. 17010261 10.1017/phn20062005

[7] World Health Organization (2014) Global Nutrition Targets 2025: Childhood Overweight Policy Brief. Geneva: WHO.

[8] WHO Multicentre Growth Reference Study Group (2006) WHO Child Growth Standards based on length/height, weight and age. Acta Paediatr Scand Suppl 450, 76–85. 10.1111/j.1651-2227.2006.tb02378.x 16817681

[9] World Health Organization (2018) WHO Global Database on Child Growth and Malnutrition. http://www.who.int/nutgrowthdb (accessed January 2018).

[10] Black RE, Allen LH, Bhutta ZA et al. (2008) Maternal and child undernutrition: global and regional exposures and health consequences. Lancet 371, 243–260. 18207566 10.1016/S0140-6736(07)61690-0

[11] Black RE, Victora CG, Walker SP et al. (2013) Maternal and child undernutrition and overweight in low-income and middle-income countries. Lancet 382, 427–451. 23746772 10.1016/S0140-6736(13)60937-X

[12] de Onis M & Blössner M (2000) Overweight prevalence and trends among preschool children in developing countries. Am J Clin Nutr 72, 1032–1039. 11010948 10.1093/ajcn/72.4.1032

[13] de Onis M, Blössner M & Borghi B (2010) Global prevalence and trends of overweight and obesity among preschool children. Am J Clin Nutr 92, 1257–1264. 20861173 10.3945/ajcn.2010.29786

[14] de Onis M, Zeitlhuber J & Martínez-Costa C (2016) Nutritional disorders in the proposed 11th revision of the International Classification of Diseases: feedback from a survey of stakeholders. Public Health Nutr 19, 3135–3141. 27293047 10.1017/S1368980016001427 PMC5217466

[15] NCD Risk Factor Collaboration (NCD-RisC) (2017) Worldwide trends in body-mass index, underweight, overweight, and obesity from 1975 to 2016: a pooled analysis of 2416 population-based measurement studies in 128·9 million children, adolescents, and adults. Lancet 390, 2627–2642. 29029897 10.1016/S0140-6736(17)32129-3 PMC5735219

[16] de Onis M & Branca F (2016) Childhood stunting: a global perspective. Matern Child Nutr 12, Suppl. 1, 12–26. 27187907 10.1111/mcn.12231 PMC5084763

[17] World Health Organization (2014) Global Nutrition Targets 2025 : Stunting Policy Brief. Geneva: WHO.

[18] World Health Organization (2018) e-Library of Evidence for Nutrition Actions (eLENA). http://www.who.int/elena/en/ (accessed January 2018).

[19] World Health Organization (2017) Guideline: Assessing and Managing Children at Primary Health-Care Facilities to Prevent Overweight and Obesity in the Context of the Double Burden of Malnutrition . Updates for the Integrated Management of Childhood Illness (IMCI). Geneva: WHO. 29578661

[20] UNICEF, World Health Organization & World Bank (2018) UNICEF–WHO–World Bank Joint Child Malnutrition Estimates. Key findings of the 2018 edition. http://www.who.int/nutgrowthdb/2018-jme-brochure.pdf?ua=1 (accessed August 2018).

[21] World Health Organization (2000) The Management of Nutrition in Major Emergencies. Geneva: WHO.

[22] World Health Organization (2014) Global Nutrition Targets 2025: Wasting Policy Brief. Geneva: WHO.

